# Studies on the Ecological Adaptability of Growing Rice with Floating Bed on the Dilute Biogas Slurry

**DOI:** 10.1155/2016/3856386

**Published:** 2016-11-01

**Authors:** Qun Kang, Rui Li, Qi Du, Bowen Cheng, Zhiqi Liao, Chengcheng Sun, Zhaohua Li

**Affiliations:** Faculty of Resources and Environmental Science, Hubei University, 368 Youyi Avenue, Wuhan, China

## Abstract

This study aimed to explore the ecological adaptability and the possibility of growing rice with floating bed on the dilute biogas slurry. The results of the experiments show that the growth stage, rice plant height, and rice yield and quality were significantly affected by multiple dilutions; rice plants cultivated with 45 multiple dilutions had better ecological adaptability than others. In the 45 multiple dilutions' group, the yield of rice was 13.3 g/bucket (8 rice plants), milled rice rate was 63.1%, and the content of crude protein in the rice was 6.3%. The concentrations of heavy metals in the rice cultivated with 30 multiple dilutions' slurry, such as total lead, cadmium, mercury, chromium, and arsenic, were all below the national standard. The study shows that it is possible and safe to cultivate rice plants with no soil but diluted biogas slurry. In the experiments, the yield, milled rice rate, and crude protein of the rice cultivated with slurry were not as much as those of rice cultivated with regular way in soil. This study provides the basic theoretical support for the development of biogas projects and the potential achievement of organic farming in special agricultural facilities and circular economy.

## 1. Introduction

Since the 21st century, energy shortage and environment pollution have become more severe. Due to the advantages in generating alternative energy and reducing environment pollution, the biogas projects developed rapidly in recent years. By the end of 2011, merely in China, 73032 biogas projects have already been established in large or medium scale [1]. For Mexico, the biogas generated from slurry has potential to produce 21 PJ per year, equivalent to 3.5% of natural gas consumption in 2013 [2]. Great attention has been paid to anaerobic digestion of animal waste, because it produces renewable energy in an environmentally friendly manner and therefore the construction of biogas plants is increasing around the world [3]. The biogas project not only reduces the agriculture waste but also produces clean bioenergy, which also contributes to considerable economic benefits.

The main problem about biogas projects is the large quantity of biogas slurry, the by-product of biogas production. Biogas slurry is a good source of plant nutrients and can improve soil properties [4–7]. The biogas slurry is normally slightly alkaline (pH 7.0–8.5). Despite the big carbon loss via methane and carbon dioxide generation, ninety percent of the raw nutrients remain in the slurry. It is estimated that the biogas slurry consists of 0.026%–0.081% total nitrogen (ammonia nitrogen 60%–75% and the rest is organic nitrogen), 0.02%–0.07% total phosphorus, and 0.047%–1.40% total potassium. In addition, biogas slurry is rich in trace elements (such as iron, zirconium, copper, boron, and molybdenum), auxins (such as gibberellin, indoleacetic acid), B group vitamins, and some antibiotics [8–10]. A tomato one-growing-season field study showed that the application of concentrated slurry can significantly bring up the total N, P, and K contents, conductivity, and fruit contents of amino acids, protein, soluble sugar, b-carotene, tannins, and vitamin C [11]. Zheng et al. found that biogas slurry-chemical fertilizers combination increased peanut grain yield and biomass, due to increases in soil N and P availability, microbial biomass C and N concentrations, and urease and dehydrogenase activities [12]. Win et al. concluded that the application of biogas slurry may be considered a substitute to the utilization of chemical fertilizer without much greenhouse gas emission and heavy metal uptake [13]. Garg et al. applied fly ash and biogas slurry combination and found positive effects on the wheat yield and soil properties [6]. From past research, we find that biogas slurry does have the potential to improve soil properties and crop yield.

However, the problem is that there is no enough land for such large quantity of biogas slurry to utilize. With the surging development of collective livestock farms, around 2 hundred million tons of biogas slurry is produced every year only in China [14]. Owing to the propagation of household-scale anaerobic digesters and biogas plants in many Asian countries, including China, the amount of biogas slurry has drastically increased [15, 16]. Large livestock farm usually does not possess enough land space for the decomposition of biogas slurry. And the excessive discharge of biogas slurry not only changes the soil property but also causes secondary pollution [17–20]. Conventional wastewater treatment is not usually adopted to treat biogas slurry, due to its high costs and waste of nutrients [21]. The utilization of such large quantity of biogas slurry is of great significance in large-scale farming and biogas energy generation. At present, biogas fertilizer is mainly used for the relatively small size of more economic crops, such as vegetables, fruits, tobacco, and aquaculture. Rice is the most important staple food crop for the demand of the increasing population of the world and about 90% of world rice production comes from Asia [22]. Due to the position of rice in China's grain production (the largest total output and acreage), rice production is undoubtedly the largest biogas fertilizer consumed in China [23]. Many investigations focus on the rice field soil properties improvement or the environment affliction by biogas slurry [13, 24]. Song et al. studied growing rice with floating beds on natural waters [25]. Tian [26] studied growing rice with floating beds on the water of reservoirs. They all got good yields. Of all the research studies, few are about the direct utilization of rice soilless cultivation. This study's aim is to explore the ecological adaptability and the possibility of growing rice with floating bed on the dilute biogas slurry.

Artificial floating bed technology [27], which belongs to a surface soilless growing technology, has been recognized as a cost-effective and feasible process in wastewater treatment, especially for decentralized wastewater in rural areas. Currently, artificial floating beds have been widely used globally and often installed over waterbodies at weirs, ponds, rivers, reservoirs, and lakes [28]. In general, artificial floating beds have followed four main functions: water purification, habitat for fish and birds, improvement of landscape, and a break-water to protect the littoral zone. The function of water purification means to effectively and permanently remove excessive nutriments (mainly N and P) out of water volumes. The amount of time required to fulfill this purpose depends on the volumes and conditions of wastewater, the extent of active biofilms, and the maturity of bed plants as well as other environmental factors. Until now, despite numerous studies on floating plantation, most efforts have focused on wastewater treatment by cultivating plants on the beds. In contrast, using biogas slurry to cultivate economic plants on the floating bed has merely been studied. Thus, more empirical studies need to be conducted and the emphasis can be put on the factors that affect the growth of floating bed species.

In order to explore the feasibility of the direct utilization of biogas slurry in soilless rice production, this study conducted a floating bed rice planting experiment with diluted biogas slurry as medium. This study has the potential to provide the theoretical support for expanding the biogas utilization processes and developing the land-, water-, and fertilizer-saving facility agriculture.

## 2. Material and Methods 

### 2.1. Materials and Planting Experiment

The planting experiment was carried out at the Hubei University, Wuhan, China, in a greenhouse with transparent rain shield cover on top.

The experimental biogas slurry was from a mesophilic biogas project in Tianmen City, Hubei Province, China. Its nutrient content and heavy metal concentration were measured. Before planting, the slurry was diluted by distilled water in descending dilution factor from 0 to 60 at intervals of 5. The diluted slurry is the medium for rice cultivation. At each dilution factor, 12-liter diluted slurry was collected and duplicates were applied. The slurry was kept into plastic buckets. 24 buckets were needed in total.

The experiment rice type is Ewan number 17. After 20 days of breeding in Tian Xin Zhou rice production area, the rice was ready for transplantation to the greenhouse. The foam board was cut into rounds with diameters slightly smaller than the buckets. Four equally spaced holes with diameters of around 2 cm were drilled on each of the foam boards. And all the foam boards were put in the buckets. Then, the rice seedlings were transplanted into the foam board of the buckets. Each small hole held two seedlings and each bucket contained eight seedlings. Seedlings were fixed with sponges. The planting experiment started on July 20th, 2015.

### 2.2. Sample Preparation and Analysis

After the seedlings were transplanted into the buckets, distilled water was added to each bucket to keep a relatively stable water volume. The slurry dilution medium in each bucket was changed every 15 days. The rice growth was observed and recorded from the beginning to the last day. The rice plants height and conductivity of the different multiple dilutions were measured. Plant height was measured with a ruler with accuracy to a millimeter, and conductivity was measured by portable conductivity meter (model: 320C-01A).

After the rice was mature, the rice panicle in each bucket was collected and the rice yield was measured. The rice husks were removed and the crude protein content and milled rice weight and rate were determined. Crude protein was measured by semimicro-Kjeldahl method. Heavy metals (copper, zirconium, lead, cadmium, and chromium) were determined by atomic absorption spectrophotometer (copper, zirconium by Flame method with model 4510; lead, cadmium, and chromium by graphite furnace method with model 4510F); Arsenic and mercury were determined by atomic fluorescence spectrometer (model: PF6-2).

### 2.3. Data Treatment and Analysis

Data are presented as mean ± standard deviation. Significant differences between and within multiple groups were examined using one-way ANOVA test followed by Tukey multiple comparisons test. The statistical analysis was performed by GraphPad Prism 6.0 software. A value of *P* < 0.05 was considered statistically significant.

## 3. Results and Analysis

### 3.1. Biogas Components and Conductivity

For nutrients, total nitrogen, phosphorus, and potassium were 0.13%, 0.023%, and 0.8% of the biogas slurry, respectively. The concentrations of the heavy metals, Zn, Cr, Cd, Pb, and Hg, were 3.33, 0.05, 0.001, 0.01, 0, and 0.004 mg/L, respectively. The pH of the slurry was 7.3 and COD_Cr_ concentration was 2,460 mg/L. The copper, zirconium, and COD_Cr_ concentrations exceeded the Chinese national standards for irrigation water quality (GB5084-1992). But when the slurry was diluted 2 times, the standards could be met.

According to Chinese national standards on the heavy metals and toxic substance in the water used in no-soil cultivation (Cu ≤ 0.1 mg/L, Zn ≤ 0.2 mg/L), the copper and zirconium concentrations exceeded the standard. When diluted 10 times, the standards could be met. In addition to the concentration of the nutrient components, either too high or too low conductivity of the culture solution used during no-soil-cultivation process can affect the growth of plants. Conductivity values of the different multiple dilutions were measured and shown in Table 1.

### 3.2. The Impact of Slurry Dilution Rate on Rice Growth Stages

The rice growth stages started from the day when it was sowed. The period of the first 20 days was a seedling stage. And, on the 20th day, rice was transplanted into the diluted slurry medium. The rice cultivated by normal way in soil usually experiences seedling stage, tillering stage, heading stage, flowering stage, grain filling stage, and fructicative stage. In the experiments, the rice cultivated with slurry of 30 to 55 multiple dilutions experienced tillering, heading, flowering, grain filling, and fructicative stages; the rice cultivated with slurry of 20, 25, and 60 multiple dilutions experienced only tillering, heading, and flowering stage. The rice cultivated with the 10 and 15 multiple dilutions' slurry experienced only tillering stage and heading stage. The relationship between the growth stages and the slurry dilution rate is shown in Table 2.

### 3.3. The Impact of Slurry Dilution Rate on Height of Rice Plants

#### 3.3.1. The Height Change Trend during Rice Cultivated in Different Multiple Dilutions

After rice was transplanted into the diluted slurry medium, the height of the plants of all groups was measured on different growing days.

**Figure 1:**
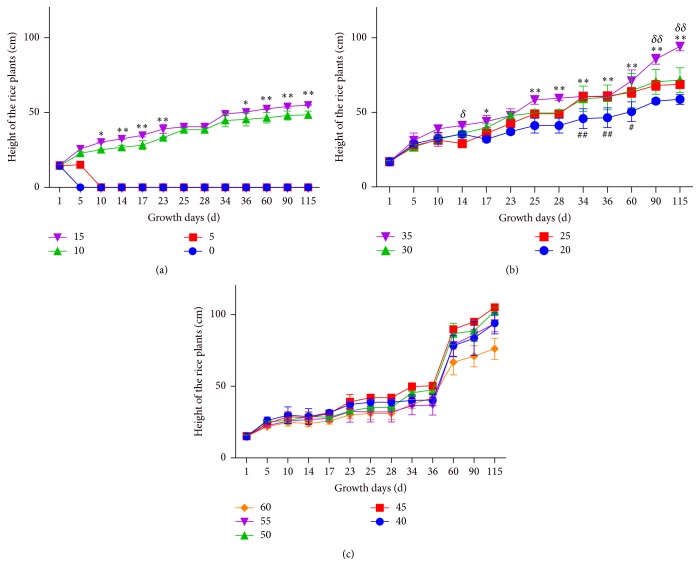
The relationship between the rice plant height and multiple dilutions during growth days. (a) represents the rice plants height of the 0, 5, 10, and 15 multiple dilutions' groups during growth days. ^*∗*^ *P* < 0.05, 15* versus* 10 multiple dilutions' group. ^*∗∗*^ *P* < 0.01, 15* versus* 10 multiple dilutions' group. (b) represents the rice plants height of the 20, 25, 30, and 35 multiple dilutions' groups during growth days, ^*∗*^ *P* < 0.05, 35* versus* 20 multiple dilutions' group, ^*∗∗*^ *P* < 0.01, 35* versus* 20 multiple dilutions' group, ^#^ *P* < 0.05, 25* versus* 20 multiple dilutions' group, ^*∗∗*^ *P* < 0.01, 25* versus* 20 multiple dilutions' group, ^*δ*^ *P* < 0.05, 35* versus* 25 multiple dilutions' group, and ^*δδ*^ *P* < 0.01, 35* versus* 25 multiple dilutions' group; (c) represents the rice plants height of the 40, 45, 50, 55, and 60 multiple dilutions' groups during growth days.

Figure 1(a) shows that the rice was alive when it was cultivated by the biogas slurry of 10 and 15 multiple dilutions in all days. On the 10th, 14th, 17th, 23rd, 36th, 60th, 90th, and 115th day, the height of rice plants cultivated by the biogas slurry of 15 multiple dilutions is greater than that cultivated by the biogas slurry of 10 multiple dilutions. On the 5th and 10th day, the rice died when cultivated by the biogas slurry of 0 and 5 multiple dilutions.

Figure 1(b) shows that the height of rice plants cultivated by the biogas slurry of 35 multiple dilutions is greater than that cultivated by the biogas slurry of 25 multiple dilutions on the 14th, 90th, and 115th day. On days 17, 25, 28, 34, 36, 60, 90, and 115, the height of rice plant which were cultivated with the biogas slurry of 35 multiple dilutions is greater than that with 20 multiple dilutions. There was no serious significance between the biogas slurries of 25 and 30 multiple dilutions in all days.

Figure 1(c) shows that all of the rice was alive cultivated with the biogas slurry of 40–55 dilutions, and the heights of rice plants have increased time-dependently.

#### 3.3.2. The Relationship between Multiple Dilutions and the Height of Rice Plant on Days 28, 60, 90, and 115

The height of plant data on the 28th, 60th, 90th, and 115th day was selected and analyzed as follows. 

**Figure 2:**
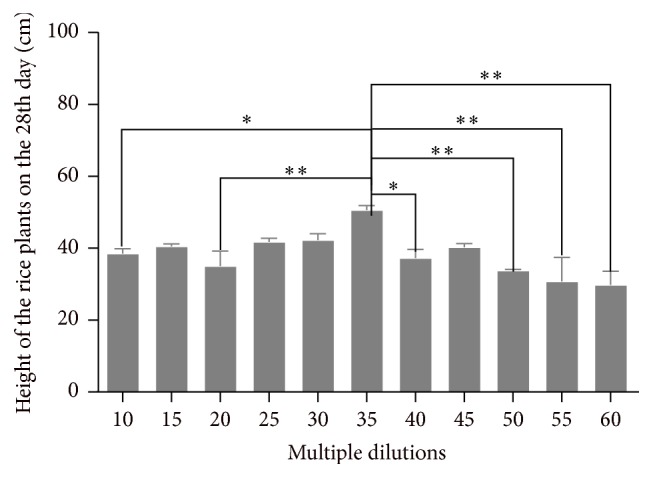
Height of the rice plant in different multiple dilutions on the 28th day. ^*∗*^ *P* < 0.05,* versus* 35 multiple dilutions' group. ^*∗∗*^ *P* < 0.01,* versus* 35 multiple dilutions' group.

*(1) The Effect of the Biogas Slurry Multiple Dilutions on the Height of Rice Plant on the 28th Day*.  Figure 2 indicates that the multiple dilutions have obvious effects on the height of rice plants in the 10–30 multiple dilutions' group on the 28th day. The height of rice plants of 35 multiple dilutions was greater than that of 10, 20, 40, 50, 55, and 60. It is not statistically significant in the 40–60 multiple dilutions' groups.

**Figure 3:**
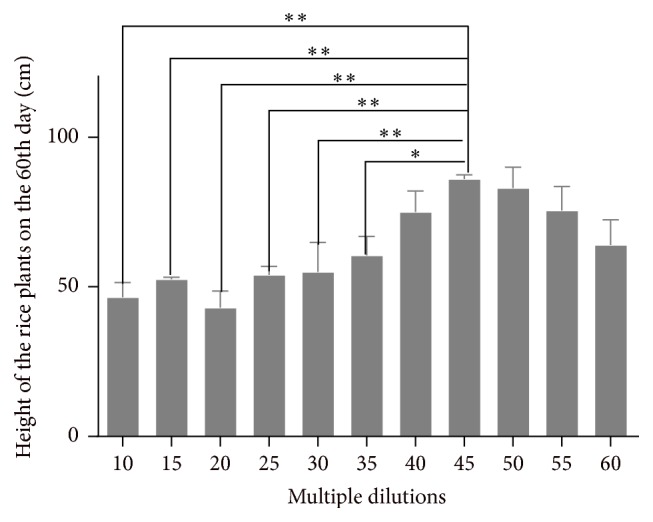
Height of rice plants in all different groups on the 60th day. ^*∗*^ *P* < 0.05,* versus* 45 multiple dilutions' group, ^*∗∗*^ *P* < 0.01,* versus* 45 multiple dilutions' group.

*(2) The Effect of the Biogas Slurry Multiple Dilutions on the Height of Rice Plant on the 60th Day*.  Figure 3 shows that there is positive relationship between 10–45 multiple dilutions of the biogas slurry and the height of rice plants, and the height of rice plants of 45 multiple dilutions was greater than that of 10, 15, 20, 25, 30, 35, and 40 multiple dilutions on the 60th day. After 45 multiple dilutions, the height of rice plants is decreased, but there is no obvious significance in 45–60 multiple dilutions.

**Figure 4:**
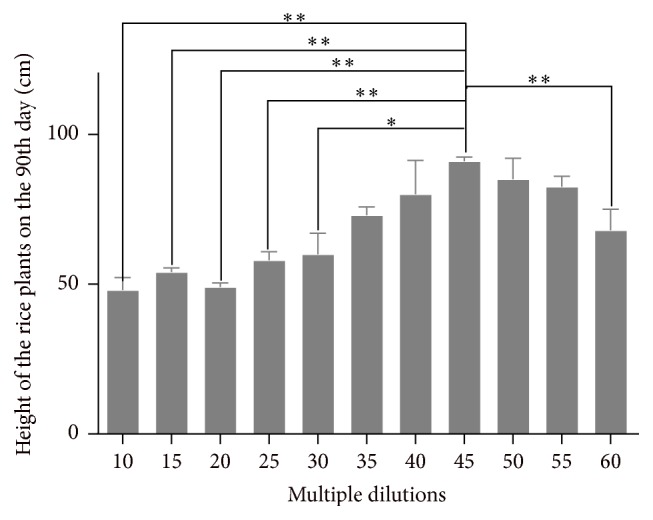
Height of rice plants in all different groups on the 90th day. ^*∗*^ *P* < 0.05* versus* 45 multiple dilutions' group; ^*∗∗*^ *P* < 0.01* versus* 45 multiple dilutions' group.

*(3) The Effect of the Biogas Slurry Multiple Dilutions on the Height of Rice Plants on the 90th Day.* Figure 4 shows that there is a direct relationship between 10–45 multiple dilutions of the biogas slurry and the height of rice plants, and the height of rice plants of 45 multiple dilutions was greater than that of 10, 15, 20, 25, 30, 35, and 40 multiple dilutions on the 90th day. After 45 multiple dilutions, there is a reverse relationship between multiple dilutions and the height of rice plants, and the height of rice plants of 60 multiple dilutions was less than that of 45 multiple dilutions. 

**Figure 5:**
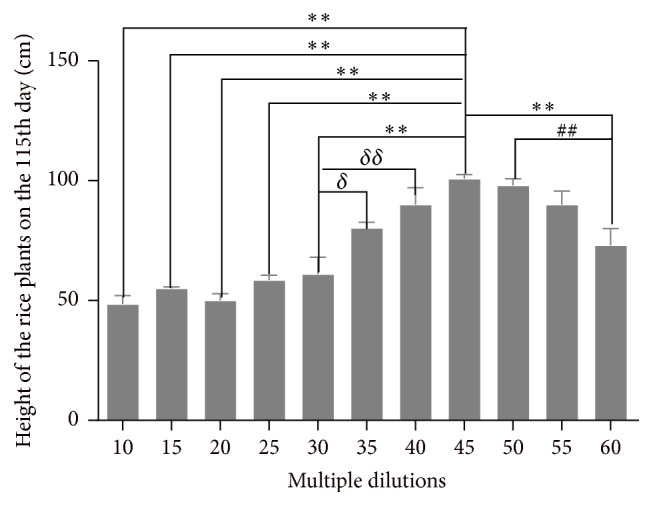
Height of rice plants in all different groups on the 115th Day. ^*∗*^ *P* < 0.05* versus* 45 multiple dilutions' group; ^*∗∗*^ *P* < 0.01* versus* 45 multiple dilutions' group; ^##^ *P* < 0.01, 50* versus* 60 multiple dilutions' group; ^*δ*^ *P* < 0.05 35* versus* 30 multiple dilutions' group; and ^*δδ*^ *P* < 0.01 40* versus* 30 multiple dilutions' group.

*(4) The Effect of the Biogas Slurry Multiple Dilutions on the Height of Rice Plants on the 115th Day.* Figure 5 shows that there is a direct relationship between multiple dilutions and the height of rice plants in 10–45 dilutions' groups on the 115th day, and, specifically, the height of rice plants of 45 multiple dilutions was greater than that of 10, 15, 20, 25, and 30, and the height of rice plants of 35 and 40 multiple dilutions was greater than that of 30. After 45 multiple dilutions, there is a reverse relationship between multiple dilutions and the height of rice plants, and the height of rice plants of 45 and 50 multiple dilutions was greater than that of 60 multiple dilutions.

Based on Figure 5, the height of rice plants cultivated with 40, 45, and 50 multiple dilutions was obviously greater than that of other dilutions on 115th day.

The height of rice plants was affected significantly by multiple dilutions; the rice was not alive in the 0–5 multiple dilutions; however, it was alive in the 10–60 multiple dilutions. The height of rice plants shows the tendency from increase to decrease with the increase of multiple dilutions, and the height of the rice plant begins to decrease after 30 dilutions on the 30th day, and it tends to decrease after 45 dilutions on the 60th, 90th, and 115th day.

When the experiment was finished on the 115th day, the height of rice plants in 40, 45, and 50 dilutions was greater than that of other multiple dilutions.

### 3.4. The Effect of the Biogas Slurry Multiple Dilutions on the Yield and Quality of the Rice

#### 3.4.1. The Effect of the Biogas Slurry Multiple Dilutions on the Yield of the Rice

**Figure 6:**
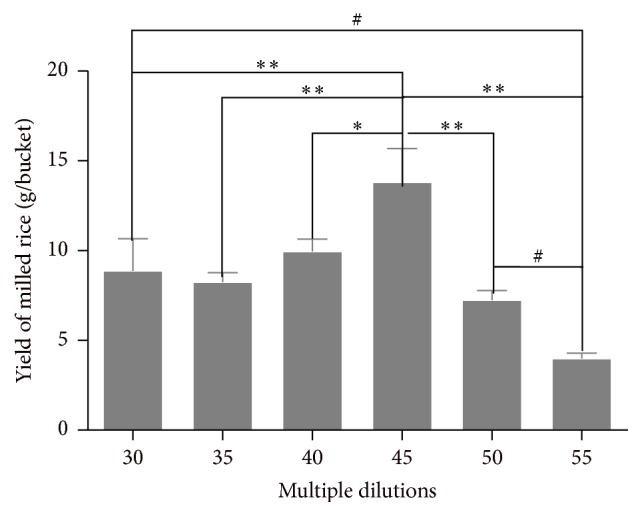
The relationship between the milled rice yield and multiple dilutions. ^*∗*^ *P* < 0.05* versus* 45 multiple dilutions' group; ^*∗∗*^ *P* < 0.01* versus* 45 multiple dilutions' group; and ^#^ *P* < 0.05* versus* 55 multiple dilutions' group.

Figure 6 shows that the rice plant produced rice with 30, 35, 40, 45, 50, and 55 multiple dilutions. There is a positive relationship between the production of rice and the multiple dilutions, and, specifically, the height in 45 multiple dilutions is the greatest one in all 30, 35, 40, and 45 multiple dilutions. There is a reverse relationship between the production of the rice and 45–55 dilutions, and moreover the production of the rice was the lowest in the 55 dilutions.

#### 3.4.2. The Effect of the Biogas Slurry Multiple Dilutions on the Content of Crude Protein in the Rice

**Figure 7:**
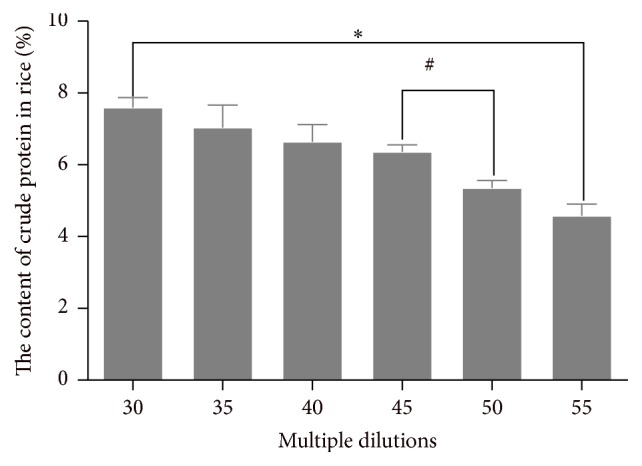
The relationship between the content of crude protein and multiple dilutions. ^*∗*^ *P* < 0.05* versus* 55 multiple dilutions' group. ^#^ *P* < 0.05* versus* 50 multiple dilutions' group.

Figure 7 shows that there is reverse relationship between the multiple dilutions and the content of crude protein in the rice. Specifically, the content of crude protein in the rice in the 30 dilutions was higher than that in 55. Moreover, there was no significant relationship between the contents of crude protein in 30, 35, 40, and 45 dilutions' groups. Finally, the content of crude protein in the 55 dilutions is the lowest.

#### 3.4.3. The Effect of the Biogas Slurry Multiple Dilutions on the Milled Rice Rate

**Figure 8:**
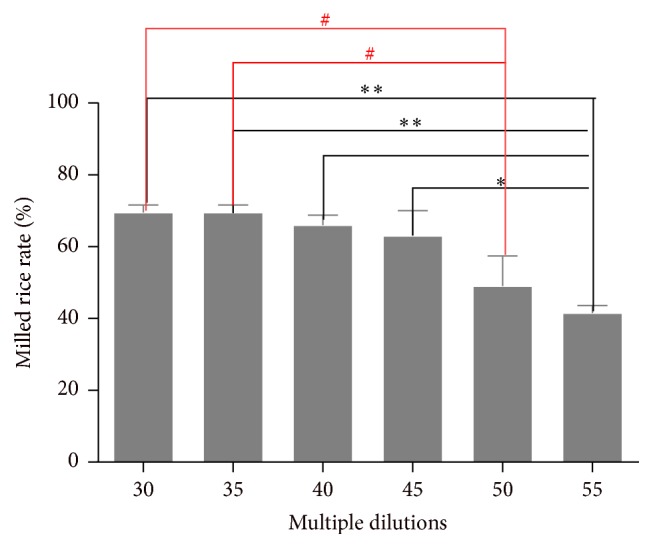
The relationship between the milled rice rate and multiple dilutions. ^*∗*^ *P* < 0.05* versus* 55 multiple dilutions' group, ^*∗∗*^ *P* < 0.01* versus* 55 multiple dilutions' group, and ^#^ *P* < 0.05* versus* 50 multiple dilutions' group.

Figure 8 shows that there was a reversed relationship between the milled rice rate and multiple dilutions. Specifically, the milled rice rates in 30, 35, 40, and 45 dilutions' groups were higher than that in 55. Moreover, the milled rice rates in 30 and 35 dilutions were higher than that in 50. Finally, there were no obviously significant relationships both in 30, 35, 40, and 45 multiple dilutions' groups and in 50 or 55 groups.

### 3.5. Heavy Metal Concentration in the Rice

The heavy metals concentrations in the rice growing in the 30 times' dilution were measured. The total lead, cadmium, mercury, and arsenic concentrations were 0.02, 0.05, 0, and 0.02 mg/kg milled rice, respectively. All reached the Chinese Hygienic Standard for Grains (GB2715-2005). Total chromium concentration was 0.05 mg/kg, which also met the requirements of the standards for food safety (GB2762-2012).

The copper and zirconium concentrations were 1.02 and 1.51 mg/kg, respectively. Both were the nutrient elements in rice.

## 4. Discussion

### 4.1. The Ecological Adaptability of Growing Rice with Diluted Biogas Slurry

The results show that the slurry dilution rate had a significant impact on the growth stages, plant height, and yield and quantity of rice. The reasons are as follows.

The plants cultivated in no soil absorb nutrition such as water and nitrogen and phosphorus and potassium elements and so on from the nutritive medium. So the nutritive medium is the key for the growth. When the dilution rate was 0 and 5, the conductivity value of the slurry was 3,262 *μ*s/cm and 2,480 *μ*s/cm, which is equal to 3/5 and 1/2 of the sea water. The inorganic salt concentration was so high that it was higher than that of plant cell fluid, which caused the death of the rice plants. When the dilution increased over 10 (the conductivity value was over 1,671 *μ*s/cm), the plants were alive and grew.

When the dilution rates were 30 to 55, the conductivity value was from 784 *μ*s/cm to 532 *μ*s/cm and the rice plants produced rice. When the dilution rate was 60 (the conductivity value was 438 *μ*s/cm) the rice plant did not grow or produce rice. The possible reason is that nutrient substance in the 60 dilutions' biogas slurry is too low. The yield of rice and height of the rice plants increased firstly and later decreased with the increase of dilutions. The possible reason is that, with the increase of dilutions, the conductivity value and nutrient substance reached a relatively suitable value for rice to grow well. But when the dilution increased, there was less of the nutrient substance in the slurry, which affects the yield of rice and height of the rice plants. Considering the situation of the yield, milled rate, and crude protein of the rice together, among all the dilutions, the 45 multiple dilutions are better than others. In the 45 multiple dilutions' group, the yield of rice was 13.3 g/bucket (8 rice plants); milled rice rate was 63.1%, which was 10% lower than that in average of rice cultivated by normal way in soil; the content of crude protein in the rice was 6.3%, which was also lower than that in average of rice cultivated by the normal way in soil (9.3%).

The concentrations of heavy metals in the rice, such as lead, chromium, and arsenate, cultivated with 30 dilutions reached the Chinese Hygienic Standard for Grains (GB2715-2005).

In this experiment, the rice yield was lower than the conventionally planted rice. The reason might be that when biogas slurry acts as the only nutrient source, the growing environment is not nutrients-balanced. When the rice fertilizer requirement increased, the biogas slurry concentration was not increased accordingly. Overall nutrients content of biogas slurry was relatively low [29]. Also the low iron content of the slurry contributed to the frequent rice iron deficiency during the experiment [30]. Regular addition of iron to the medium could maintain a stable growth of lettuce, celery, tomato, cucumber, and eggplant. This indicated that the addition of chelated iron was effective in solving biogas slurry iron shortage [31].

The heavy metals' concentrations in the rice growing in the 30 times' dilution were measured. The total lead, cadmium, mercury, and arsenic concentrations reached the Chinese Hygienic Standard for Grains (GB2715-2005). It shows cultivating rice in over 30 dilutions' biogas slurry is safe.

### 4.2. Swine Manure-Biogas Slurry-Rice-Biofuel Circle Mode

**Figure 9:**
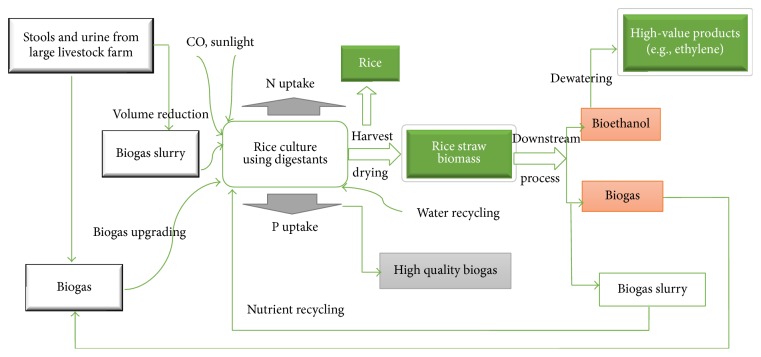
Swine manure-biogas slurry-rice-biofuel production circle.

Scalable rice production with biogas slurry not only can bring economic benefits by harvesting rice but also can provide an economic production model with swine manure-biogas slurry-rice-biofuel biomass energy circle (Figure 9). As shown in Figure 7, rice could be obtained by the cultivation in biogas slurry, while rice straw could be used for the generation of biofuels such as bioethanol and biogas. The by-product of rice straw fermentation was biogas slurry, which can in turn be used to grow rice. Thus, a nitrogen circle was formed by this approach. The process also presented a highly viable measure for biogas upgrading, since CO_2_ from crude biogas was consumed during the microalga photosynthesis. The production of straw-based biofuels in the form of bioethanol and biogas can compensate energy consumed in the system [32]. In other words, the biofuels generated can serve as the primary energy source for the operation of the whole process.

## 5. Conclusions

This study shows that it is possible and safe to cultivate rice plants on the floating bed with no soil but the diluted biogas slurry from by-products of biogas project. Among 0–60 multiple dilutions, rice plants cultivated with 45 multiple dilutions had better ecological adaptability than others. However the yield, crude protein, and milled rice rates of the rice plants cultivated with 45 multiple dilutions are not as much as those of rice cultivated in the regular way in soil.

The next research direction is comparing the nutrition components in the diluted biogas slurry with the nutrition components in the regular soil and adding extra nutrients or compound fertilizer to the biogas slurry in different rice growth stages for the purpose of increasing the rice yield and quantity.

This technique provides a problem solving approach to the big amount of slurry discharge in the livestock farms. Apart from economic benefits, the production circle of swine manure-biogas slurry-rice-biofuel has the potential to contribute to reducing the carbon footprint and protecting the environment.

## Figures and Tables

**Table 1:** The conductivity value of different multiple dilutions biogas slurry (*μ*s/cm).

Multiple dilutions	0	5	10	15	20	25	30	35	40	45	50	55	60
Conductivity (*μ*s/cm)	3262	2480	1671	1192	974	872	784	719	638	605	566	532	438

**Table 2:** The growth stages of rice cultivated with different diluted biogas slurry.

Multiple dilutions	Duplication	Tillering stage	Heading stage	Flowering stage	Grain filling stage	Fructicative stage
10	1	√	√	×	×	×
	2	√	√	×	×	×

15	1	√	√	×	×	×
	2	√	√	×	×	×

20	1	√	√	√	×	×
	2	√	√	√	×	×

25	1	√	√	√	×	×
	2	√	√	√	×	×

30	1	√	√	√	√	√
	2	√	√	√	√	√

35	1	√	√	√	√	√
	2	√	√	√	√	√

40	1	√	√	√	√	√
	2	√	√	√	√	√

45	1	√	√	√	√	√
	2	√	√	√	√	√

50	1	√	√	√	√	√
	2	√	√	√	√	√

55	1	√	√	√	√	√
	2	√	√	√	√	√

60	1	√	√	√	×	×
	2	√	√	√	×	×

Note: √ shows the rice plants have ever experienced the growth.

× shows the rice plants have not experienced the growth.
